# Tianma Gouteng Decoction combined with Qiju Dihuang Pill for the treatment of essential hypertension: A protocol for systematic review and meta-analysis

**DOI:** 10.1097/MD.0000000000021157

**Published:** 2020-07-17

**Authors:** Xuan Chen, Lijuan Li, Xiangmei Xu, Wenna Yang, Jie Wang, Yixuan Kong, Jinghui Zheng

**Affiliations:** a Graduate School, Guangxi University of Chinese Medicine; b Department of Geriatrics, Ruikang Affiliated Hospital of Guangxi University of Chinese Medicine, Nanning, Guangxi, China.

**Keywords:** essential hypertension, meta-analysis, Qi Ju Di Huang Wan, Tianma Gouteng Decoction

## Abstract

**Background:**

Essential hypertension is one of the risk factors of cardiovascular and cerebrovascular diseases, which can cause target organ damage such as heart, brain and kidney, and has extremely high disability rate and death rate. With the development of economy and society, the prevalence rate of hypertension in China has increased rapidly from 9.8% in the 1980s to over 30% in the 21st century. According to the data published in “China Cardiovascular Disease Report 2018,” China currently has 245 million hypertension patients. Comprehensive prevention and treatment of hypertension has become one of the major public health problems in China. The clinical practice and theoretical innovation of traditional Chinese medicine in the prevention and treatment of hypertension have been carried out for decades. Relevant literature points out that Tianma Gouteng Decoction combined with Qiju Dihuang Pill has ideal effect in the treatment of primary hypertension. However, most of the literatures are small sample studies, with uneven quality and clinical evidence, and lack of evidence-based medical evidence for clinical efficacy. Therefore, this study makes further meta-analysis of Tianma Gouteng Decoction combined with Qiju Dihuang Pill in the treatment of primary hypertension, with a view to providing evidence-based medical evidence for the treatment of primary hypertension.

**Methods:**

We will search 3 foreign electronic databases (Cochrane Library, Embase, PubMed) and 4 Chinese electronic databases (China National Knowledge Infrastructure, WangFang Database, Chinese Biomedical Literature Database, and Chinese Scientific Journal Database) to collect potential systematic reviews from their inceptions to February 2020. The language of publication is limited to Chinese or English. First, the quality of randomized controlled trials documents included in this study was evaluated by using the improved Jadad scoring scale. Then, the 2 researchers conducted the evaluation independently according to Cochrane bias risk tools. The evidence level of the results will be evaluated by using the recommended evaluation, development and evaluation grading of recommendations assessment, development, and evaluation method. Statistical analysis will be conducted using Revman 5.3.

**Results:**

The results of this study will be published in a peer-reviewed journal.

**Conclusions:**

The conclusion of this study will provide evidence for the efficacy of Tianma Gouteng Decoction combined with Qiju Dihuang Pills in the treatment of primary hypertension due to the efficacy of western medicine alone in treating primary hypertension.

**Registration number PROSPERO:**

INPLASY202050088.

## 1 Introduction

Essential hypertension is one of the risk factors of cardiovascular and cerebrovascular diseases, which can cause target organ damage such as heart, brain and kidney, and has extremely high disability rate and death rate. With the development of economy and society, the prevalence rate of hypertension in China increased rapidly from 9.8% in 1980s to over 30% in 21st century.^[1]^ According to the data published in “China Cardiovascular Disease Report 2018,” China currently has 245 million hypertension patients,^[2]^ Comprehensive prevention and treatment of hypertension has become one of the major public health problems in China. Research shows that compared with the United States, the prevalence rate of hypertension in China is lower than that in the United States, but the average blood pressure is significantly higher than that in the United States, and the awareness rate, control rate, and treatment rate are low. The increased burden of hypertension in China may be the cause of the high incidence of stroke in Chinese brain.^[3]^ Essential hypertension is the most common cardiovascular disease, and the onset age is more common among the elderly.^[4]^ Essential hypertension, as a common cardiovascular disease, has the characteristics of “3 low” (low treatment rate, low control rate, and low awareness rate) and “3 no” (no discomfort, no medication, no tolerance, and irregular medication), which seriously affects people's quality of life.^[5]^ The pathogenesis of hypertension is complex and cannot be completely cured, but reasonable and effective treatment measures can control blood pressure, reduce complications of hypertension, and improve the quality of life of patients with hypertension.

In recent years, with the development of social economy, the aging of the population, changes in eating habits, and other factors, the prevalence of hypertension has been increasing. Hypertension belongs to the categories of “head wind,” “wind dizzy,” and “vertigo” in the concept of traditional Chinese medicine. Its causes are mostly caused by overwork, improper diet, unsmooth emotions, and excessive physique, which promote phlegm and blood stasis in viscera, endogenous wind and fire, imbalance of qi activity, disorder of qi and blood, imbalance of yin and yang.^[6]^ The disease mainly includes 4 types: phlegm-dampness type, qi and blood deficiency type, liver yang hyperactivity type and kidney essence deficiency type, and liver yang hyperactivity type is more common in clinic.^[7]^ Tianma Gouteng Decoction is composed of Rhizoma Gastrodiae, Ramulus Uncariae cum Uncis, Concha Haliotidis, Fructus Gardeniae, Scutellariae Radix, Radix Cyathulae, Eucommiae Cortex, Herba Leonuri, Herba Taxilli, Caulis Polygoni Multiflori, and Poria with hostwood. It has the effects of calming the liver, calming the wind, clearing heat, promoting blood circulation, and tonifying liver and kidney. It is a good agent for calming the liver and lowering adverse qi. It is commonly used in clinical treatment of hypertension, cerebrovascular disease, ear-derived vertigo and other syndromes of wind-yang disturbance and liver-yang hyperactivity.^[8]^ Qiju Dihuang Pill is made from Liuwei Dihuang Pill plus Fructus Lycii and Flos Chrysanthemi. Fructus Lycii is sweet, smooth and moist, enters lung, liver and kidney meridians, tonifies kidney and essence, nourishes liver and improves eyesight. Chrysanthemum: pungent, bitter, sweet, slightly cold, good at clearing and benefiting the head, dispersing heat of liver meridian, calming liver, and improving eyesight. the combination of the 8 drugs can jointly play the roles of nourishing yin, nourishing liver and improving eyesight, and is especially effective for patients with yin deficiency of liver and kidney with hypertension accompanied by obvious head and eye diseases such as dizziness and blurred vision.^[9]^ He Xiufen^[10]^ Clinical Application of Tianma Gouteng Decoction Ophiopogon japonicus, Rehmannia glutinosa, and Scrophularia ningpoensis are added for patients with eye astringency and obvious tinnitus; The total effective rate in the treatment group was 92.16%, significantly higher than that in the control group. Li Guanping^[11]^ Clinical application of Qi Ju Di Huang Wan combined with Tian Ma Gou Teng Yin in the treatment of 97 cases of primary hypertension showed that the effect of Qi Ju Di Huang Wan combined with Tian Ma Gou Teng Yin in the clinical treatment of patients with primary hypertension was significant. Huang Xiaoling et al^[12]^ have applied Qiju Dihuang Pill combined with Tianma Gouteng Decoction in the treatment of 94 cases of primary hypertension. The results show that Qiju Dihuang Pill combined with Tianma Gouteng Decoction has ideal blood pressure control effect and high safety in the treatment of primary hypertension.

At present, hypertension patients need to take antihypertensive drugs for a long time, but taking antihypertensive drugs for a long time will not only lead to various adverse reactions of patients, but also lead to continuous reduction of patient compliance. The clinical practice and theoretical innovation of traditional Chinese medicine in the prevention and treatment of hypertension have been carried out for decades. Relevant literature points out that Tianma Gouteng Decoction combined with Qiju Dihuang Pill has ideal effect in the treatment of primary hypertension. However, most of the literatures are small sample studies, with uneven quality and clinical evidence, and lack of evidence-based medical evidence for clinical efficacy. Therefore, this study makes further meta-analysis of Tianma Gouteng Decoction combined with Qiju Dihuang Pill in the treatment of primary hypertension, with a view to providing evidence-based medical evidence for the treatment of primary hypertension.

## 2 Methods

### 2.1 Study registration

This protocol was recorded in the International Platform of Registered Systematic Review and Meta-analysis Protocols (INPLASY), registration number INPLASY202050088 (https://inplasy.com/inplasy-2020-5-0088/). And if there are any changes, we will describe it in our full review.

### 2.2 Inclusion criteria for study selection

#### 2.2.1 Type of studies

The randomized controlled trial (RCT) of Tianma Gouteng Decoction combined with Qiju Dihuang Pill in the treatment of essential hypertension is limited to Chinese and English.

#### 2.2.2 Type of participants

In line with the diagnostic criteria for essential hypertension, the patient's sex, age, race, onset time, and source of cases are not limited.

#### 2.2.3 Type of interventions

Tianma Gouteng Decoction combined with Qiju Dihuang Pills. The specific traditional Chinese medicines include: Tianma, Uncaria, Poria, Chuanxiong, Alisma orientale, Lycium barbarum, Corni Fructus, Eucommiae Cortex, Scutellariae Radix, Gardenia Fructus, Cyathula officinalis, Chrysanthemum, Haliotidis, and so on.

#### 2.2.4 Type of comparator (s)/control

The control group was treated with conventional western medicine.

#### 2.2.5 Types of outcome measurements

Systolic blood pressure; diastolic blood pressure; clinical total effective rate; adverse reaction.

#### 2.2.6 Search methods for identification of studies

We will search 3 foreign electronic databases (Cochrane Library, Embase, PubMed) and 4 Chinese electronic databases (China National Knowledge Infrastructure, WangFang Database, Chinese Biomedical Literature Database, and Chinese Scientific Journal Database) to collect potential systematic reviews from their inceptions to February 2020. The language of publication is limited to Chinese or English. The following search terms will be used: Tianma Gouteng Decoction, Tianma Gouteng Decoction, Qiju Dihuang Pill, Hypertension, Hypertension, Essential Hypertension, and so on.

#### 2.2.7 Selection of studies

The 2 researchers independently sifted through the literature, extracted the data, and cross-checked them. In case of disagreement, they will settle it through discussion or third-party negotiation. When selecting documents, first read the title. After excluding obviously irrelevant documents, further read the abstract and the full text to determine whether to include them. If necessary, contact the original research author via email or telephone to obtain uncertain but very important information for this research. The contents of data extraction include:

(1) the basic information of the research of the NaRen: the research topic, the first author, and the publication time;
(2) Baseline characteristics of the study subjects such as gender, age, intervention measures, and treatment time of the study subjects;
(3) the key elements of the improved Jadad score;
(4) outcome indicators and outcome measurement data of concern.

**Figure 1:**
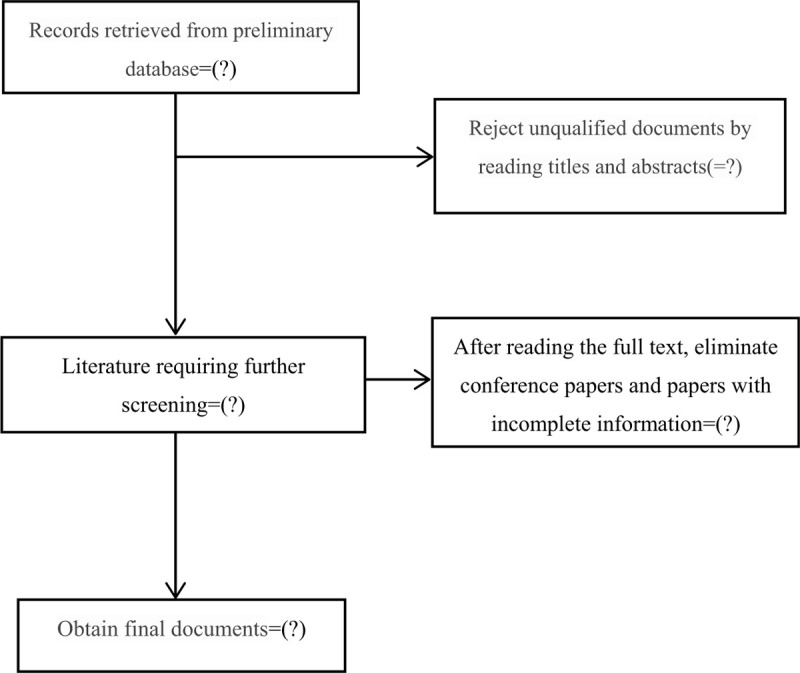
Flowchart of literature selection.

The planned selection pricess is shown in a flow chart (Fig. 1).

#### 2.2.8 Literature quality evaluation

All the documents included in the study were evaluated with the improved Jadad rating scale^[13]^ for the quality of RCT documents included in the study. The improved Jadad rating scale includes 4 aspects: random sequence generation, randomization concealment, blind method, and withdrawal and withdrawal. The first 3 aspects are divided into appropriate, unclear, and inappropriate 3 grades, with appropriate score of 2 points, unclear score of 1 point, inappropriate score of 0 point, and withdrawal and withdrawal aspects. A score of 1 is given for describing the number and reasons of withdrawal and withdrawal. A score of 0 is given for unspecified number and reasons of withdrawal and withdrawal. A full score of 7 is given. Documents greater than or equal to 1 can be included in this study. A score of 1 to 3 is considered as low quality and a score of 4 to 7 is considered as high quality.

#### 2.2.9 Assessment of reporting bias

Two researchers independently evaluated the risk of bias included in the study and cross-checked the results. The bias risk assessment tool uses the RCT bias risk assessment tool provided by Cochrane Collaboration Network,^[14]^ which includes 7 aspects of random allocation method, hidden allocation scheme, blind method application, integrity of result data, selective reporting of research results and other bias sources. The bias risk assessment is divided into 3 levels: low risk, unclear risk, and high risk.

#### 2.2.10 Statistical processing of literature data

In this study, the RCT documents included in this study were statistically processed using Review Man5.3 software provided by Cochrane Collaboration Network. The results of dichotomous variables are represented by odds ratio and the results of continuous variables are represented by mean difference. When the homogeneity of the results included in each study is good, that is, *P* > .05, *I*^2^ < 50%, a fixed model is used; when the homogeneity of the results included in each study is poor, that is, *P* < .05, *I*^2^ > 50%, a random model is used.

#### 2.2.11 Grading the quality of evidence

According to the recommendation of grading of recommendations assessment, development, and evaluation tool,^[15]^ the evidence quality evaluation of key result indicators can be divided into 4 levels: high (+++), medium (++), low (++), and very low (+).

## 3 Ethics and dissemination

Ethical approval and the informed consent are not required for this research is not the clinical study and personal information is not involved.

## 4 Discussion

Hypertension in traditional Chinese medicine belongs to the category of “vertigo” and “headache.” Vertigo was first seen in Neijing from Suwen to Zhenda Yaolun, which states that “all wind falls dizzy, which belongs to liver”; Lingshu Weiqi, which states that “upper deficiency leads to dizziness”; and Lingshu Hailun, which states that “deficiency of marrow sea leads to tinnitus in brain.” Huangdi Neijing believes that vertigo is mainly caused by deficiency, mainly in liver and kidney, mainly due to imbalance of yin and yang, qi and blood, deficiency of kidney qi, water does not contain wood, wood is not nourished, liver yin is damaged, and liver yang is hyperactive, which leads to dizziness. The main factors leading to the onset of essential hypertension are arteriosclerosis, the thickness of blood vessel wall of patients with muscle enlargement, reduction of elasticity, and reduction of blood vessel inner diameter, thus causing serious insufficiency of blood supply.^[16]^ Hypertension can lead to endothelial dysfunction and accelerate the occurrence and development of cardiovascular and cerebrovascular diseases.^[17]^ The key to clinical treatment of essential hypertension lies in continuous control of blood pressure stability.^[18]^ Modern Chinese medicine research believes that “liver and kidney imbalance” is an important pathogenesis of primary hypertension.^[19]^ With the development of social economy, the aging of the population, changes in eating habits, and other factors, the prevalence rate of hypertension is increasing. At present, hypertension has become one of the important public health problems in our country.^[20]^ Qi Ju Di Huang Wan and Tian Ma Gou Teng Yin are traditional drugs for treating hypertension.^[21]^ Tianma Gouteng Yin comes from Hu Guangci's “New Meaning of Treating Miscellaneous Diseases” in modern times. It has the effects of calming the liver, calming the wind, clearing heat and promoting blood circulation, and tonifying the kidney. Qiju Dihuang pill belongs to Chinese patent medicine. It is based on Liuwei Dihuang pill and has the effects of nourishing liver and kidney, nourishing yin and improving eyesight. In this study, through meta-analysis of RCT research on the treatment of primary hypertension by Tianma Gouteng Decoction combined with Qiju Dihuang Pills, the clinical practice and theoretical innovation of traditional Chinese medicine in the prevention and treatment of hypertension have been for decades. Relevant literature points out that Tianma Gouteng Decoction combined with Qiju Dihuang Pills has ideal effect on the treatment of primary hypertension, but most of the literatures are small sample studies, with uneven quality and clinical evidence quality and lack of evidence-based medical evidence for clinical efficacy. Therefore, this study makes further meta-analysis on the treatment of primary hypertension by Tianma Gouteng Decoction combined with Qiju Dihuang Pills in order to provide evidence-based medical evidence for the treatment of primary hypertension.

## Author contributions

**Conceptualization:** Xuan Chen.

**Data curation:** Xuan Chen, WenNa Yang.

**Formal analysis:** LiYing Lu, XiangMei Xu.

**Investigation:** YiXuan Kong, Jie Wang.

**Methodology:** LiJuan Li, Xuan Chen, XiangMei Xu.

**Software:** YiXuan Kong, LiJuan Li.

**Writing – original draft:** LiJuan Li, Xuan Chen, WenNa Yang, XiangMei Xu, YiXuan Kong, Jie Wang.

**Writing – review & editing:** LiJuan Li, Xuan Chen, WenNa Yang, XiangMei Xu.
